# Ischemic Heart Disease in Patients with Inflammatory Bowel Disease: Risk Factors, Mechanisms and Prevention

**DOI:** 10.3390/life12081113

**Published:** 2022-07-24

**Authors:** Alina Ecaterina Jucan, Otilia Gavrilescu, Mihaela Dranga, Iolanda Valentina Popa, Bogdan Mircea Mihai, Cristina Cijevschi Prelipcean, Cătălina Mihai

**Affiliations:** 1 Saint Spiridon County Hospital, 700111 Iași, Romania; alina-ecaterina_ghiata@email.umfiasi.ro (A.E.J.); bogdan.mihai@umfiasi.ro (B.M.M.); cristina.cijevschi.prelipcean@umfiasi.ro (C.C.P.); catalina.mihai@umfiasi.ro (C.M.); 2 Faculty of Medicine, University of Medicine and Pharmacy “Grigore T. Popa”, 700115 Iași, Romania; iolanda-valentina.g.popa@umfiasi.ro

**Keywords:** ischemic heart disease, inflammatory bowel disease, ulcerative colitis, Crohn’s disease, myocardial infarction, coronary artery disease, cardiovascular risk

## Abstract

According to new research, a possible association between inflammatory bowel disease (IBD) and an increased risk of ischemic heart disease (IHD) has been demonstrated, but this concern is still debatable. The purpose of this review is to investigate the link between IHD and IBD, as well as identify further research pathways that could help develop clinical recommendations for the management of IHD risk in IBD patients. There is growing evidence suggesting that disruption of the intestinal mucosal barrier in IBD is associated with the translocation of microbial lipopolysaccharides (LPS) and other endotoxins into the bloodstream, which might induce a pro-inflammatory cytokines response that can lead to endothelial dysfunction, atherosclerosis and acute cardiovascular events. Therefore, it is considered that the long-term inflammation process in IBD patients, similar to other chronic inflammatory diseases, may lead to IHD risk. The main cardiovascular risk factors, including high blood pressure, dyslipidemia, diabetes, smoking, and obesity, should be checked in all patients with IBD, and followed by strategies to reduce and manage early aggression. IBD activity is an important risk factor for acute cardiovascular events, and optimizing therapy for IBD patients should be followed as recommended in current guidelines, especially during active flares. Large long-term prospective studies, new biomarkers and scores are warranted to an optimal management of IHD risk in IBD patients.

## 1. Introduction

Inflammatory bowel disease (IBD) is a recurrent chronic idiopathic inflammatory condition of the gastrointestinal tract. Various factors are involved in its pathogenesis, such as genetic susceptibility of the host, and it is precipitated by environmental and microbial factors [1]. Crohn’s disease (CD) and ulcerative colitis (UC) are the two major subtypes of IBD, characterized by chronic intestinal inflammation, while the most common symptoms are frequent diarrhea, often with blood and pus in stools, abdominal pain and cramping, fever, and weight loss [2]. The incidence and prevalence of IBD are still increasing worldwide. Besides of the primary gastrointestinal complications of IBD, a broad-spectrum of extra-intestinal manifestations and IBD complications have also been outlined due to persistent long-standing systemic inflammation [3,4].

Ischemic heart disease (IHD) is still the leading global cause of death worldwide; there is a general concern to identify patients with cardiovascular risk factors and to apply preventive measures.

The state of chronic inflammation in IBD can lead to endothelial dysfunction and platelet aggregation, confers a higher risk of developing atherosclerosis and coronary artery disease, and thereby a higher risk of acute coronary events [5]. IBD patients have been highlighted to have increased carotid intimal thickness, endothelial dysfunction, and wall stiffness, mainly due to increased circulating inflammatory cytokines [6]. Thus, several inflammatory mediators such as high C-reactive protein (CRP) and circulating pro-inflammatory markers such as tumor necrosis factor-α (TNF-α) and interleukins are involved in the pathogenesis of IBD, as well as in atherosclerosis [7]. Increased levels of the aforementioned inflammatory mediators, together with increased burden of traditional cardiovascular disease risk factors in the general population, drive a higher risk of IHD in IBD patients [8].

Multiple large population studies have shown a positive association between IBD and IHD, especially in women and young patients, but the data remain controversial [9,10]. The pathophysiological mechanisms behind this phenomenon have not been fully understood. We speculate that the difference between IBD and non-IBD men regarding IHD risk becomes estompated due to higher prevalence of traditional cardiovascular risk factors in men compared to women. Furthermore, higher risk of acute arterial events observed in younger IBD patients may reflect the different impact of inflammation across age groups. The use of contraceptive pills and higher CRP levels among women could also be a contributory factor.

## 2. Epidemiological Links between IBD and IHD

We searched PubMed utilizing the keywords: “inflammatory bowel disease”, “IBD”, “guidelines”, “treatment plan”, “ischemic heart disease”, and “diagnosis” in all possible combinations. We extracted information regarding diagnosis, management, and treatment linking the two diseases, IBD and IHD. This review aims to summarize the current knowledge related to IBD and IHD with respect to its pathophysiology and risk factors in order to promote further research that can improve understanding and help develop clinical practice guidelines for prevention and management of IHD in patients with IBD.

### Epidemiology

Large cohort studies evaluated the link between IBD and risk of IHD (Table 1) and found conflicting results.

Although some retrospective cohort studies did not find significant associations [11,15,18,23], the meta-analysis [13,14,19] found a positive correlation between IBD and IHD. In a meta-analysis conducted in 2017 by Feng et al. [19] were included 10 cohort studies investigating the risk of developing IHD in IBD. Researchers noticed an elevated risk of developing IHD in IBD patients compared to matched controls without IBD (RR = 1.244). Data found women, young age (<50 years), short-term follow-up (<5 years) may be at high IHD risk [19]. A large population trial conducted by Panhwar et al. in the United States of America in 2019 [22], involved a large database over 29 million patients from 26 different healthcare systems nationwide. A higher prevalence of acute MI was observed in both patients with UC and CD, as compared to non-IBD patients (the frequency of MI- UC 6.7% vs. CD 8.8% vs. non-IBD 3.3%, odds ratio [OR] for UC 2.09 [2.04–2.13] and CD 2.79 [2.74–2.85]), but the risk of having an acute cardiovascular event was highest in younger IBD patients (30–34 years old) and decreased with age (OR 12.05 [11.16–13.01]). The reasons for this issue have not been fully established – the higher CRP concentrations and the use of contraceptive pills among women could be decisive [22].

On the other hand, various studies have shown that the risk of myocardial injury mortality is lower among patients diagnosed with IBD when compared with non-IBD patients [21,23]. This can be attributed to the protective role of currently therapy of IBD based on 5-ASA, thiopurines and biologic therapy used in this patient group as part of their treatment. Salicylic acid shows anti-inflammatory and anti-oxidant properties, which may suggest a cardio-protective effect of 5-ASA when used prolonged, and a decreased risk of IHD compared to patients who have never received 5-ASA [12]. TNF-α blockers present reliable anti-inflammatory properties, and a number of studies are now available to report their protective effect on the risk of IHD in patients treated with anti-TNF drugs [24].

The retrospective study conducted by Barnes et al. showed that patients with IBD were less likely to be admitted to hospital for acute myocardial injury compared to general population (1.3% vs. 3.2%; *p* < 0.001); in the adjusted analysis of risk factors for IHD, the OR was 0.54 for patients with IBD hospitalized for MI [18]. In 2021, another interesting study published by Sinh et al. aimed to investigate the MI outcomes in 2,629,161 patients, of which 3784 with CD and 3607 with UC. It showed that IBD did not impact in-hospital mortality due to MI-UC (odds ratio [OR], 1.12; 95% CI 0.98–1.29) and CD (OR 0.99; 95% CI 0.86–1.15). However, patients diagnosed with UC had higher total hospitalization costs compared to patients with MI without IBD [23].

Despite the fact that more recent attention has been paid to the possible links between IHD and IBD, some issues remain uncertain and the results are still unclear. Nevertheless, clinicians need to consider screening for IHD in all patients with IBD with a particular focus on women with IBD and younger adults (under the 50 years of age), who appear to be at the highest risk of developing an acute myocardial injury.

## 3. Risk Factors for IHD in IBD Patients

### 3.1. Traditional Cardiovascular Risk Factors

Traditional cardiovascular risk factors associated with IHD are obesity, type 2 diabetes mellitus (DM), hypertension, hyperlipidemia, smoking, and stress [25]. Some of them (Western lifestyles, chronic stress, tobacco in CD) are present in both diseases.

Classically, patients with IBD are considered underweight due to malnutrition. However, with the increasing prevalence of obesity in the general population and the emergence of innovative therapies that control and maintain remission in IBD, the prevalence of obesity can reach 40% of patients with IBD [26]. Obesity increases thromboembolic risk, the risk of surgery in UC, the perianal damage, and the need for hospitalization in CD [27]. However, Hu’s [28] meta-analysis demonstrates that obese patients with IBD have a better evolution compared to non-obese patients, with a lower probability of hospitalization, surgery, and corticosteroid therapy.

Large population studies show an increased risk of type 2 DM in patients with IBD, independent of corticosteroid use [29]. There are few studies that prospectively follow the evolution of IBD in patients with DM. Published data suggest increased inflammatory activity, increased resource requirements, decreased QoL, increased risk of complications, infections, and higher mortality in diabetic patients with IBD [30].

Both metabolic syndrome and IBD have an increasing incidence and prevalence, as a consequence of lifestyle changes, with the widespread adoption of the “Western” type. The association of IBD with metabolic syndrome is not accidental, as there are common etiopathogenic links between the two diseases: inflammation, abnormal immune response, disorders in the endocrine function of adipose tissue, intestinal dysbiosis [31].

In a recent study, Golovics et al. identified older age, female gender, hyperlipidemia, and hypertension (*p* < 0.001 for each) as risk factors for developing MI in both CD and UC in the logistic-regression-based prevalence models. DM has also been labelled as an additional risk factor for MI in both CD and UC [32]. In a large database, Panhwar et al. examined the risk of MI in patients with or without IBD, and noted that traditional cardiovascular risk factors were more common among patients with both UC and CDIBD and MI [22]. On the other hand, the association between IBD and the high risk of MI persisted despite adjustments for traditional cardiovascular risk factors, thus suggesting that IBD may represent an independent risk factor for developing MI [22].

The study conducted by Correia et al. [33] revealed that a high percentage of women that used oral contraceptive pills (OCPs) had an elevated risk of MI. The use of hormonal contraception is associated with the risk of developing acute cardiovascular events, correlated to the pro-inflammatory state of IBD. This could possibly explain the increased risk of acute coronary syndrome in young women with IBD.

### 3.2. Risk Factors Related to IBD

#### 3.2.1. Increased IHD with Disease Activity

Disease activity may have an independent impact on the risk of acute arterial events in patients with IBD. Le Gall, et al. [20] demonstrated that clinically active IBD was significantly associated with an increased risk of acute ischemic events in patients with IBD (Odds ratio (OR): 12.3, 95%CI: 2.8 ± 53.6). The disease activity was evaluated trough indirect markers, including hospitalizations, surgical treatments, and exposure therapies. Additionally, as reported in the Danish study, the risk of cardiovascular events is highest during active flares; this risk decreases during times of remission [9]. Periods of active flares (defined as 3-month periods before and after IBD-related hospitalization or surgery) were independently associated with an elevated risk of cardiovascular events in patients with CD (HR 1.74, 95% CI 1.44–2.09) and patients with UC (HR 1.87, 95% CI 1.58–2.22) [10]. Card et al. conducted a cohort analysis of the association between IBD, disease activity and the risk of MI, stroke and cardiovascular mortality. Although they did not find a significant increase in vascular events in patients with IBD in general, the study demonstrated that the incidence of the events correlated with a higher disease activity [34]. Furthermore, Agca et al. revealed in their study that cardiovascular events occur especially during disease flares in undertreated patients [35].

The activation of the coagulation cascade and proinflammatory cytokines as a consequence of active intestinal inflammation may be a factor that contributes to the occurrence of acute arterial events [36]. Disease activity should be regarded as a modifiable risk factor for cardiovascular events, and aggressive control of inflammation might reduce the risk of thrombosis in patients with IBD.

#### 3.2.2. IBD Treatment

Corticosteroids are used in the management of acute flares of IBD, and as mentioned above, several studies have demonstrated an increased risk of IHD in acute flares and the fulminant and active stages of IBD. There are inconsistent data on whether corticosteroids have an increased cardiovascular risk in IBD patients, and thus it is difficult to decipher whether the increase in cardiovascular events during this time period is due to the direct effect of steroids or the uncontrolled disease activity. The adverse effects of long-term steroid use in IBD patients were studied by Lewis et al. in their cohort study [37]. CD patients had increased mortality with prolonged steroid use as compared with anti-TNF use; that was mainly related to major cardiovascular events (nonfatal MI, nonfatal stroke, and need for vascularization) [37]. Furthermore, in their article, Close et al. revealed that patients with UC had a higher incidence of IHD and MI with steroid use [16].

In the study conducted by Jaaouani et al. [38] the use of aminosalicylates, immune modifiers, and biologic therapies did not affect acute coronary syndrome events. However, exposure to anti-TNFs is associated with a decreased risk of acute arterial events in patients with IBD, particularly in men with CD [39]. A study conducted by Paschou et al. [40] revealed a decrease in insulin levels and homeostatic model assessment for insulin resistance index in patients with IBD after receiving treatment with biological therapy for a period of six months. Data suggest that clinical treatment can promote not only controlling intestinal inflammation, but also controlling risk factors for cardiovascular disease, resulting in the reduction of the overall risk of cardiovascular events in the long term [41]. However, prospective studies are needed to prove these effects in the general IBD population. With the advent of new drugs that enable better control of inflammatory activity and the establishment of treatment strategies with defined therapeutic targets, a reduction and a better control of the cardiovascular risk in IBD population is expected.

## 4. Inflammation—The Main Pathogenic Links between IBD and IHD

It is known that inflammation has been involved in the pathogenesis of atherosclerosis and coronary artery disease. Elevated markers of inflammation are associated with increased cardiovascular risk in all patients, with or without an inflammatory disorder [42].

IBD is associated with deregulation and increase in various cytokines [43]. Pro-inflammatory markers like homocysteine and CRP, which are known to be increased in patients with cardiovascular disease, are also found in chronic systemic inflammation in conditions like IBD [35]. CRP is a predictor of cardiovascular events and may contribute to atherogenesis [44]. The serum CRP level greater than 5mg/L during one year or in the previous 3 years were all associated with an higher risk of acute ischemic event (OR: 3.2, 95%CI: 1.2 ± 8.5) [20]. It has been established that its concentration increases in an active phase of IBD, thus proving that the cardiovascular risk is higher when CRP concentrations are increased.

**Figure 1:**
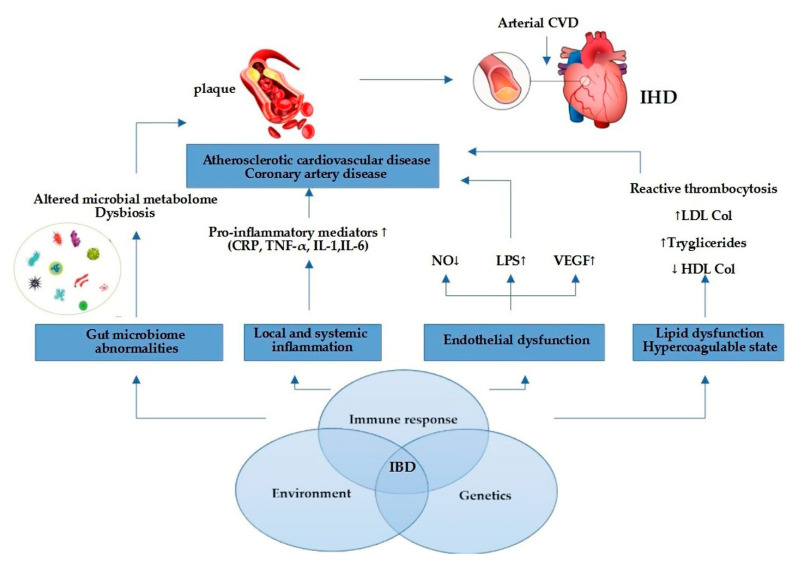
Link between IBD and IHD. Elevated pro-inflammatory mediators promote atherosclerotic plaque formation and cardiovascular events through endothelial dysfunction, gut microbiome abnormalities, pro-inflammatory state, and lipid dysfunction. Abbreviations: NO = nitric oxide; LPS = lipopolysaccharide; VEGF = vascular endothelial growth factor; LDL = low-density lipoprotein; HDL = high-density lipoprotein; CRP = C-reactive protein; TNF-α = tumor necrosis factor alpha; IL-1 = interleukin-1; IL-6 = interleukin-6; CVD = cardiovascular disease; IHD = ischemic heart disease.

As mentioned in Figure 1, other representatives pro-inflammatory mediators involved in IBD are tumor necrosis factor alpha (TNF-α), immunoglobulins (IgG, IgM), interleukin-6 (IL-6), interleukin-1 (IL-1), and vascular endothelial growth factor (VEGF). Notably, TNF-α is a proatherogenic cytokine [45] because blockade of TNF-α with biological therapy (infliximab, adalimumab) diminished severity of UC or CD [46] and enhanced the endothelial dysfunction in IBD patients [47]. VEGF, which is known to promote vessel formation, may contribute to IBD by increasing angiogenesis and inflammation [48]. Activation of these cytokines can increase oxidative stress, endothelial dysfunction, and macrophage accumulation, which can also stimulate atherosclerotic plaque formation [49]. Chronic inflammation promotes structural and functional changes of the endothelium. It has been acknowledged that the disrupted intestinal mucosal barrier in IBD facilitates the translocation of microbial lipopolysaccharides (LPS) and other endotoxins into circulation, inducing expression of pro-inflammatory cytokines [50], which may contribute to endothelial damage, atherosclerosis, and cardiovascular events. The mechanism that highlights this link has not been well established, but the state of chronic inflammation is considered to have a significant contribution for both IBD and IHD progression [51]. Other probable mechanisms involved could be arterial stiffening and coronary microcirculatory dysfunction [52]. In recent research, which included 17 studies with 558 UC patients, and 693 CD patients, the correlation between arterial stiffness and IBD was explored, and showed that the strength of the association of arterial stiffness between UC and CD was similar [53]. The association between IBD and arterial stiffness enhanced the hypothesis of systemic inflammation, possibly playing a role in the pathogenesis of arterial stiffness, which is widely recognized as a crucial intermediate process of CVD [53]. Furthermore, the dysfunctional endothelial system in patients with UC and CD has been observed as the markers of endothelial function.

At the molecular level, it has been showed that the increased expression of Toll-like receptors 2 and 4 (TLR2 and 4) in inflammatory cells likely mediate the damaging signaling events triggered by LPS and other microbial toxins, and in fact, elevated levels of TLR2 and 4 have been observed in atherosclerotic plaques [49].

In addition, under conditions of chronic inflammation, phenotypical changes in vascular smooth muscle occur, as well as medial calcification and reduced elasticity of vessels [49].

More than that, changes in nutrition and absorption, next to inflammation, can lead to lipid alterations.

Chronic systemic inflammation has been shown to be implicated in all phases of IHD, from vascular endothelial dysfunction to the onset and rupture of atherosclerotic plaque [54]. Increased permeability is clearly present in IBD, and could be a hypothesis leading to abnormal absorption of bacteria and toxic substances from the intestinal microbiota, which drives, as a consequence, both enteric and systemic inflammatory reactions and the diffusion in the bloodstream of bacterial endotoxins [55]. Inflammatory cytokines and modified lipoproteins are also largely responsible for the increased production of reactive oxygen species, which increases the expression of cell adhesion molecules by stimulating leukocyte migration to the subendothelial space, a key component in initiating and maintaining the atherosclerotic process [56,57].

Nevertheless, chronic inflammation, characteristic for IBD and associated with increased concentrations of pro-inflammatory cytokine represent the essential factor associated with the severity of IBD.

## 5. Proposed Strategies for IHD Prevention among IBD Patients

### 5.1. Traditional Cardiovascular Risk Factors Modification in IBD Patients

Cardiovascular prevention should be started soon after the diagnosis of IBD as the highest risk is in the first years of evolution [58,59]. Optimal management involves the multidisciplinary team, together with the patient, according to evidence-based interventions, in order to reduce the risk of IHD [60]. All patients with IBD should be screened for cardiovascular risk factors identification; their presence requires aggressive management. Screening includes lifestyle habits, smoking status, body mass index, blood pressure, glucose, and lipid profile [61]. Stratification of cardiovascular risk in IBD patients is a challenge, as the scores used in the general population are difficult to translate into a young population. Complete tobacco cessation is key.

The statins’ role in cardiovascular prevention in IBD patients is not fully understood. Patients with IBD typically have normal lipid levels, although some studies have reported alterations in lipid profile, especially HDL-cholesterol [5]. In addition to the lipid-lowering and stabilizing effect of atheroma plaque, statins also have anti-inflammatory properties [62]. The study conducted by Lochhead et al. revealed that statin treatment may have a protective role in the onset of CD, regardless of age, sex, comorbidities, or type of statin [63]. However, until further high-quality prospective research focusing on the role of statins in IBD progression should be performed, the role of statins in preventing IBD is still limited, conflicting, and has important limitations [63]. Until then, statins will be used according to the same rules as in the general population, with the mention that the presence of IBD is an enhancer for initiating therapy.

### 5.2. Disease Activity Control

Inflammation is the main trigger in IHD development in IBD patients. Cardiovascular disease especially occurs during disease flares in undertreated patients. Therefore, it is necessary to optimize the management of IBD, especially during active flares. IBD therapy not only controls intestinal inflammation, but also has the potential to prevent cardiovascular events in these patients [64]. Aminosalicylates and anti-TNF agents may decrease cardiovascular risk, while corticosteroids increase it [10]. Deep remission is an ultimate treatment goal in the management of patients. New treatment drug options may provide expectations for long-term remission with lower relapse rates.

The main guidelines recommendations regarding the management of IHD risk in IBD patients are resumed in Table 2.

## 6. Conclusions

Systemic inflammation in IBD patients leads to oxidative stress and elevated levels of inflammatory cytokines such as TNF-α, leading to phenotypic changes in smooth muscle cells that culminate in atherosclerosis and CVD. The significance of IBD in causing atherosclerosis, ischemic heart disease and myocardial infarction is currently being recognized.

Patients with IBD are at increased risk of IHD—particularly women and young patients with IBD flare. The management of IBD patients should focus on a multidisciplinary, team-based approach to preventive care, remission of IBD disease activity, and aggressive reduction of cardiovascular risk factors, and thus gastroenterologists and cardiologists should work together to screen for cardiovascular risk factors and optimize anti-inflammatory treatment in IBD patients. Future prospective studies are needed to understand common etiopathogenic mechanisms, to find biomarkers and scores for patient stratification, and to establish optimal management.

## Figures and Tables

**Table 1:** Studies evaluating the risk of ischemic heart disease in inflammatory bowel disease patients.

Author	Year of Publication	Type of Study	Study Showing the Association between IBD and IHD	Conclusion
Osterman et al. [11]	2011	Retrospective cohort study	No	IBD patients did not appear to be at elevated risk of early MI when compared with patients from general practice.
Rungoe et al. [12]	2013	Cohort study	Positive	People diagnosed with IBD were compared with IBD-free individuals during 1997–2009 (*n* = 28,833). The risk of IHD was highest in the first year after IBD diagnosis (IRR = 2.13). The risk of IHD was 1.22 during 1–13 years of follow-up after IBD diagnosis.
Kristensen et al. [9]	2013	Cohort study	Positive	IBD patients had an increased total risk of MI (RR, 1.17 [95% confidence interval 1.05–1.31]). During periods of persistent IBD activity the RRs of MI increased to 1.49 (1.16–1.93). In remission periods, the risk of MI was similar to controls.
Fumery et al. [13]	2014	Meta-analysis	Positive	The study found an increased risk of IHD (RR, 1.23; 95% CI, 0.94–1.62). Cardiovascular mortality in patients with IBD compared to general population was not increased.
Singh et al. [14]	2014	Meta-analysis	Positive	There has been a modest increase in the risk of CV morbidity due to IHD, particularly in women.
Ruisi et al. [15]	2015	Cohort study	No	The study did not show an association with IBD and premature CV events in a cohort of 300 patients with IBD without traditional risk factors for CV disease.
Close et al. [16]	2015	Retrospective cohort study	Positive	A higher proportion of IBD patients were diagnosed with IHD: 2220 (11.6%) compared with 6504 (8.6%) of controls. Most IHD diagnoses predated the diagnosis of IBD. Patients with UC had a higher risk of IHD (unadjusted HR 1.3 (95% CI 1.1–1.5), *p* < 0.001) or MI (unadjusted HR 1.4 (95% CI 1.1–1.6), *p* = 0.004).
McAuliffe et al. [17]	2015	Retrospective cohort study	Positive	Patients with moderate to severe IBD had increased rates of MI vs. patients with mild IBD.
Barnes et al. [18]	2016	Retrospective cross-sectional study	No	Patients with IBD demonstrated lower rates of acute MI than in the general population (1.3% vs. 3.1%, *p* < 0.001).
Feng et al. [19]	2017	Meta-analysis	Positive	Increased risk of IHD in IBD patients (RR, 1.244; 95% CI, 1.142–1.355). Increased risk in CD (RR, 1.243; 95% CI, 1.042–1.482) compared to UC (RR, 1.206; 95% CI, 1.170–1.242).
Le Gall et al. [20]	2018	Cohort Study	Positive	Occurrence of AAE (acute coronary syndrome). Disease activity may increase the risk of AAE.
Sun et al. [21]	2018	Meta-analysis	Positive	Higher risk of MI in women with IBD than in men; inflammation seems to play a more important role in CV disease in women than in men.
Kirchgesner et al. [10]	2018	Cohort study	Positive	IBD patients are at increased risk of AAE—SIR 1.35, with the highest risk in young patients.
Panhwar et al. [22]	2019	Cohort study	Positive	The prevalence of MI was higher in patients with UC and CD than in patients without IBD (UC 6.7% vs. CD 8.8% vs. non-IBD 3.3%). The relative risk of MI was associated with a higher rate in younger patients, and decreased with age.
Sinh et al. [23]	2021	Retrospective cross-sectional study	No	The study showed no difference between in-hospital mortality in patients with MI with or without UC (7.75% vs. 7.05%; *p* = 0.25) or in patients with MI with or without CD (6.50% vs. 6.59%; *p* = 0.87). Patients with MI with IBD had a longer length of stay.

Abbreviations: IBD—inflammatory bowel disease; MI—myocardial infarction; RR—rate ratio; UC—ulcerative colitis; CD—Crohn’s disease; IHD—ischemic heart disease; CV—cardiovascular; SIR—standardised incidence ratio; AAE—acute arterial events.

**Table 2:** Guidelines statements regarding IHD risk in IBD patients.

Guideline	Recommendation
ECCO, 2015 [65] The First European Evidence-based Consensus on Extra-intestinal Manifestations in Inflammatory Bowel Disease.	The risks of IHD, cerebrovascular accident, and mesenteric ischaemia are modestly increased in IBD, particularly in women Systemic inflammation predisposes to premature atherosclerosis Cardiovascular mortality has not been shown to be increased in IBD
International consensus on the prevention of venous and arterial thrombotic events in patients with inflammatory bowel disease, 2021 [66].	Epidemiology - It is an increased risk of arterial thrombosis in young patients - IBD female patients have an increased risk of stroke and IHD compared to males Traditional cardiovascular risk factors should be screened and controlled in all IBD patients The risk of both arterial and venous thrombotic events is increased during IBD flares. Disease activity control is one of the main factors of cardiovascular protection IBD Therapy - 5-ASA (long term administration) and Anti-TNF agents decrease the risk of arterial thrombosis and IHD - Steroids increase both arterial and venous thrombotic events in IBD patients
2019 ESC Guidelines for the diagnosis and management of chronic coronary syndromes [67].	Lifestyle changes, smoking cessation, maintaining an optimal body weight, a healthy diet, and regular exercise are the most important measures in preventing cardiovascular risk. Patients with IBD, along with those with other inflammatory conditions (systemic lupus erythematosus, rheumatoid arthritis) and neoplasms have an additional cardiovascular risk; they require aggressive screening, prevention and management measures
2019 ACC/AHA guidelines [68].	For initiating or intensifying statin therapy in adults with borderline and intermediate-risk for atherosclerotic cardiovascular disease, inflammatory diseases are “risk-enhancing” clinical factors

Abbreviations: ECCO—European Crohn’s and Colitis Organisation; IHD—Ischemic heart disease; IBD—Inflammatory bowel disease; 5-ASA—5-aminosalicylic acid; ESC—European Society of Cardiology; Anti-TNF—Anti-Tumor Necrosis Factor; ACC/AHA—American College of Cardiology/American Heart Association.

## Data Availability

Not applicable.
